# Crosstalk between endothelial cells and osteoblasts stimulates ALP via Notch signaling and RANKL/OPG ratio independently of Notch signaling in vitro

**DOI:** 10.1186/s11658-025-00793-9

**Published:** 2025-10-02

**Authors:** Katharina Wirsig, Nina Bürger, Anne Bernhardt

**Affiliations:** Centre for Translational Bone, Joint- and Soft Tissue Research, Faculty of Medicine and University Hospital, TU Dresden, Fetscherstraße 74, 01307 Dresden, Germany

**Keywords:** Osteoblasts, Endothelial cells, ALP, Notch, RANKL/OPG ratio

## Abstract

**Background:**

Bone remodeling requires a complex interplay between osteogenesis and angiogenesis, orchestrated by yet not fully understood intricate signaling pathways in osteoblasts and endothelial cells.

**Methods:**

In the present study, co-cultures of primary human osteoblasts and human umbilical vein endothelial cells (HUVEC) were compared with osteoblast cultures treated with dexamethasone (Dex), vascular endothelial growth factor (VEGF), their combination, or VEGF in the presence of Notch inhibitor N-[N-(3,5-difluorophenacetyl)-l-alanyl]-S-phenylglycine t-butyl ester (DAPT). Cellular behavior was analyzed at morphological, gene expression, and protein levels to identify key regulators in the interplay between osteoblasts and endothelial cells.

**Results:**

Dex and VEGF additively increased alkaline phosphatase (ALP) in osteoblast–HUVEC co-cultures, but not in osteoblast cultures. Furthermore, Dex reduced the receptor activator of nuclear factor κB ligand/osteoprotegerin (RANKL/OPG) ratio in osteoblasts. This effect was reversed in the presence of VEGF, but only in co-culture, indicating a direct action of endothelial cells, rather than VEGF itself, in stimulating RANKL and reducing OPG in osteoblasts. In addition, Notch signaling, specifically NOTCH1 and DLL4, was induced in response to VEGF solely in co-cultures. The presence of Notch inhibitor DAPT suppressed VEGF-induced stimulation of ALP but not RANKL/OPG ratio.

**Conclusions:**

Our findings provide novel evidence for the significant role of endothelial cells in bone remodeling, specifically in regulating ALP expression and activity of osteoblasts via the Notch signaling pathway and RANKL/OPG ratio independent of Notch. This study underscores the applicability and significance of multicellular tissue models for studying bone turnover processes in vitro, thereby reducing the reliance on animal testing.

**Graphical abstract:**

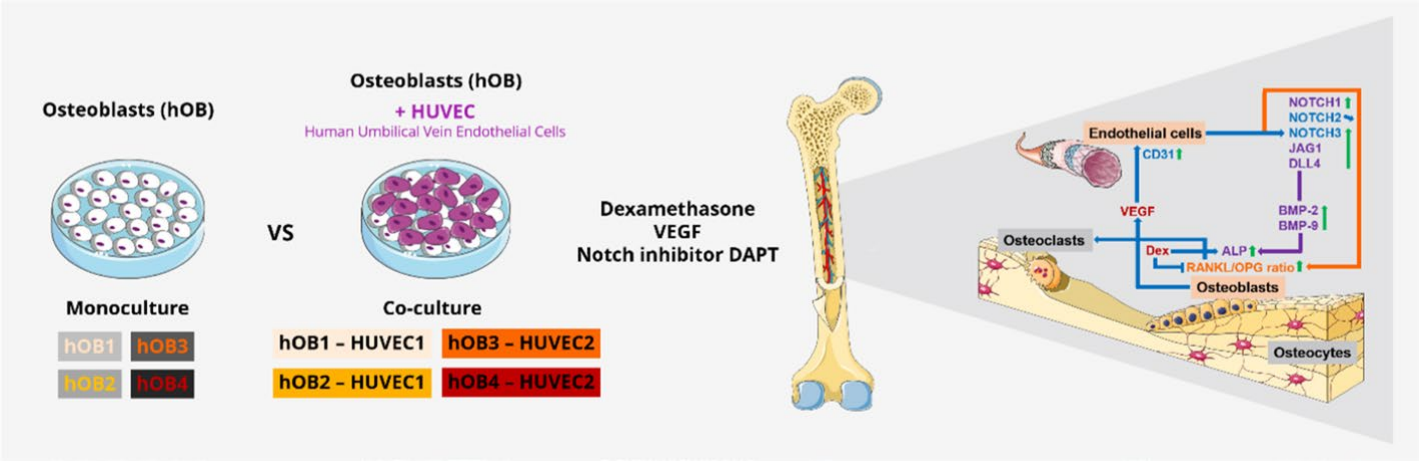

**Supplementary Information:**

The online version contains supplementary material available at 10.1186/s11658-025-00793-9.

## Introduction

Bone is a vascularized tissue that undergoes constant remodeling. This process is facilitated by bone-forming osteoblasts (OBs) and bone-resorbing osteoclasts under the control of sensing osteocytes [1]. Osteogenesis and angiogenesis are mutually dependent processes in bone remodeling and repair [2, 3]. Blood vessels are essential for maintaining bone homeostasis by supplying oxygen and nutrients, removing waste products and transporting inorganic calcium and phosphate ions necessary for calcification [4]. Therefore, in vitro bone models should include both bone cells and endothelial cells to closely mimic this tissue. In vitro models are applicable to recapitulate key cellular interactions, molecular signaling pathways, interactions between different cell types, and microenvironmental factors in a controlled and reproducible manner without the ethical concerns of animal testing. Many previous studies have focused on in vitro bone models, most of them using immortalized cell lines such as osteoblast-like MG-63 cells [5] or MC3T3-E1 cells [6] instead of primary human cells, thereby constraining the accuracy of their models compared with human in vivo processes. Furthermore, the lack of including endothelial cells, which are crucial for vascular supply and interaction, in most bone models, highlights the limitations of in vitro bone modeling approaches. To overcome these limitations, this study applied primary human OBs (hOBs) and human umbilical vein endothelial cells (HUVECs) in direct co-culture to investigate cellular interactions between bone cells and endothelial cells in comparison with hOB monocultures. The integration of endothelial cells provides a more comprehensive and accurate representation of bone development, remodeling, and repair processes.

Vascular endothelial growth factor A (VEGF-A) is a well-known angiogenic growth factor. Stimulation of endothelial cells with VEGF induces gene expression related to cell survival, growth, migration, and invasion into the surrounding tissue and formation of tube-like structures [7]. VEGF is essential for the coupling of osteogenesis and angiogenesis. As a chemotactic molecule, VEGF attracts endothelial cells toward bone tissue and directly controls the differentiation and functions of OBs and osteoclasts [8]. OBs [8] and osteocytes [9] secrete VEGF, which underlines the close link between osteogenesis and angiogenesis. In vivo, VEGF production increases after tissue damage or inflammation and it promotes healing processes, including bone repair. Disruption of the vascular network can lead to localized hypoxia, inducing the hypoxia-inducible factor (HIF) signaling pathway, which in turn stimulates VEGF secretion [10]. Invasion of blood vessels and the targeted migration of growth factors, immune cells, and osteogenic progenitor cells in response to VEGF are essential for bone regeneration [11].

Dexamethasone (Dex), a synthetic member of the glucocorticoid family, is used as an anti-inflammatory and antiallergic drug owing to its immunosuppressive effects [12]. Unfortunately, Dex has a number of side effects, including the induction of osteoporosis. Glucocorticoids stimulate receptor activator of nuclear factor κB ligand (RANKL) expression in OBs, which leads to an activation of osteoclasts [13]. However, the exact cellular mechanisms of glucocorticoid-induced osteoporosis are not fully understood. Binding of RANKL to its receptor RANK, located on the surface of osteoclast precursor cells, initiates the differentiation and activity of osteoclasts. Osteoprotegerin (OPG), the counteracting protein, inhibits osteoclastogenesis when it binds to RANKL.

To gain insight into the crosstalk between OBs and endothelial cells, as well the mechanisms by which Dex and VEGF act on bone metabolism, hOBs and HUVECs were treated in direct co-cultures with Dex, VEGF, the combination of Dex and VEGF, or VEGF in the presence of the Notch inhibitor N-[N-(3,5-difluorophenacetyl)-l-alanyl]-S-phenylglycine t-butyl ester (DAPT) (γ-secretase inhibitor) for 14 days in comparison with hOB cultures. Cellular behavior was analyzed at morphological, gene expression, and protein levels to identify key regulators in the interplay between OBs and endothelial cells.

## Materials and methods

### Cell culture

Primary human pre-OBs were isolated from human femoral heads from patients with osteoarthritis undergoing total hip replacement at the University Hospital Carl Gustav Carus Dresden (Germany) after informed consent (approval by the Ethics Commission of TU Dresden), as previously described [14]. Bernhardt et al. referred to the pre-OB fraction as “control cells” in this article. Pre-OBs were expanded in proliferation medium (alpha minimum essential medium (α-MEM) with GlutaMAX (Gibco) containing 10% fetal calf serum (FCS) and 100 U/mL penicillin and 100 µg/mL streptomycin (PS) (Gibco)) until passage 4. To prove OB character, isolated pre-OB were checked for alkaline phosphatase (ALP) activity after osteogenic differentiation for 14 days in passage 2.

HUVECs were purchased from Promocell (cat. no. C-12205; lot no. 489Z003, 487Z020.1) and expanded in endothelial cell growth medium (EGM, Promocell) until passage 3.

**Fig. 1:**
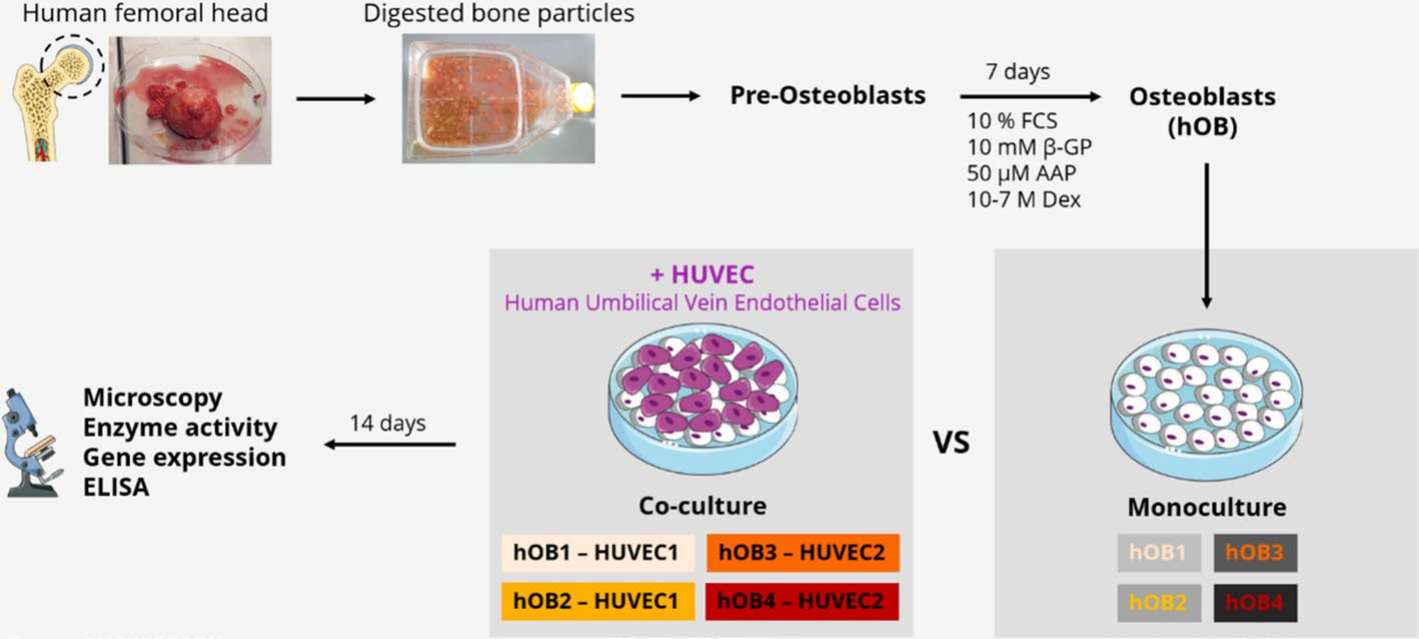
Experimental setup of hOB monocultures in comparison with direct hOB–HUVEC co-cultures. OBs were isolated from human femoral heads, expanded, and osteogenically pre-differentiated. OB monocultures and OB–HUVEC co-cultures were conducted over 14 days. Analyses included fluorescence microscopy, ALP activity measurements, gene expression analysis, and RANKL, OPG, and VEGF enzyme-linked immunosorbent assay (ELISA). This Figure includes modified illustrations obtained from Servier Medical Art. β-GP, β-glycerophosphate; AAP, ascorbic acid-2-phosphate; Dex, dexamethasone; hi-FCS, heat-inactivated FCS; hOBs human osteoblasts

For osteogenic differentiation, human pre-OBs in passage 4 were cultured in osteogenic medium (proliferation medium supplemented with 10^–7^ M dexamethasone (Dex), 10 mM β-glycerophosphate (β-GP), and 12.5 µg/mL ascorbic acid-2-phosphate (AAP), all osteogenic supplements from Sigma-Aldrich) for 7 days (Fig. 1). Subsequently, hOBs were seeded in 48-well plates (1 × 10^4^ per well) and cultured in proliferation medium for 3 days. For hOB–HUVEC co-cultures, HUVECs in passage 4 were seeded onto the hOB layer in 48-well plates (1 × 10^4^ per well). Both direct co-cultures and hOB cultures were cultivated for 14 days in seven different media (Table 1), with media changes twice a week. No cytotoxic effects of the Notch inhibitor DAPT up to a concentration of 50 µM on hOB and HUVEC were observed using the commercially available CellTiter-Glo^®^ Luminescent Cell Viability Assay (Promega) (Supplementary Information: Supplementary Fig. S1). All tested media contained the osteogenic supplements AAP and β-GP to maintain osteogenic differentiation (Tab. 1).

**Table 1:** Media compositions for hOB monocultures and hOB–HUVEC co-cultures

Medium	Supplements
Control (Ctr) Dex Dex VEGF10 Dex VEGF20 VEGF10 VEGF20 VEGF20 DAPT	50% α-MEM, 50% EBM, 2% hi-FCS, 1% PS, 5 mM β-GP, 12.5 µg/mL AAP Control medium, 10 nM Dex Control medium, 10 nM Dex, 10 ng/mL VEGF Control medium, 10 nM Dex, 20 ng/mL VEGF Control medium, 10 ng/mL VEGF Control medium, 20 ng/mL VEGF Control medium, 20 ng/mL VEGF, 40 µM DAPT

Direct hOB–HUVEC co-cultures in comparison with hOBs were analyzed in six independent experiments with four different donor combinations, involving four hOB donors and two HUVEC donors: hOB1 (female 75 years)—HUVEC1; hOB2 (male 57 years)—HUVEC1; hOB3 (male 65 years)—HUVEC2; hOB4 (female 62 years)—HUVEC2. Information on donor age of the HUVECs, purchased from PromoCell, was not provided.

### Fluorescence microscopy

For high-quality fluorescence microscopy images, hOBs and hOB–HUVEC co-cultures were performed on chamber slides (ibidi µ-slides 8-well). After washing (phosphate-buffered saline (PBS)), fixation (4% formaldehyde in PBS), permeabilization (0.1% Triton X-100 in PBS for 5 min, followed by washing with PBS), and blocking (1% bovine serum albumin (BSA) in PBS for 30 min), hOB–HUVEC co-cultures were incubated overnight with 1 µg/mL mouse anti-human CD31 (Dako; M0823). After washing, they were incubated for 1 h with 1 µg/mL DAPI, Phalloidin-iFluor 488 (Abcam), and 4 µg/mL AlexaFluor 546 goat anti-mouse IgG (Invitrogen; A11030). hOB cultures were incubated with DAPI/Phalliodin-iFluor488 accordingly. Z-stack images were captured using a Keyence BZ-X810 fluorescence microscope. Image processing was performed with ImageJ (Fiji).

### Alizarin red staining

Alizarin red staining was applied for the detection of calcium deposits, indicating mineralization and mature osteoblast differentiation. Therefore, after the 14-day cultivation, hOBs in monoculture and hOB–HUVEC co-cultures in 8-well ibidi µ-slides were fixed with 4% formaldehyde in PBS for 1 h and, after washing with distilled water, incubated in 2% alizarin red dissolved in distilled water (pH 4.1–4.3; Sigma-Aldrich) for 3 min. After washing with distilled water, brightfield images were captured using a Keyence BZ-X810 microscope. Image processing was performed using ImageJ (Fiji).

### RNA isolation, cDNA synthesis, and polymerase chain reaction (PCR)

RNA was isolated from six technical replicates (hOB–HUVEC co-cultures or hOB monocultures) of each experimental group using the commercially available peqGOLD MicroSpin Total RNA Kit (Peqlab). Lysates of three samples were pooled to finally obtain two RNA samples per group.

For cDNA synthesis, the High-Capacity cDNA Reverse Transcription Kit (Applied Biosystems) was applied according to the manufacturer’s instructions.

Gene expression analysis was performed using quantitative (q)PCR reactions with the TaqMan Fast Advanced Master Mix (Applied Biosystems) and TaqMan Gene Expression Assays for the following genes: actin-β (*ACTB*; Hs01060665_g1); receptor activator of NF-κB Ligand (*RANKL/TNFSF11*; Hs00243522_m1); osteoprotegerin (*OPG/TNFRSF11B*; Hs00900358_m1); alkaline phosphatase (*ALPL*; Hs01029144_m1); platelet endothelial cell adhesion molecule (*PECAM1/CD31*; Hs01065279_m1); vascular endothelial growth factor (*VEGFA*; Hs00900055_m1); neurogenic locus Notch homolog protein 1 (*NOTCH1*; Hs01062014_m1); neurogenic locus Notch homolog protein 2 (*NOTCH2*; Hs01050702_m1); neurogenic locus Notch homolog protein 3 (*NOTCH3*; Hs01128537_m1); delta-like canonical Notch ligand 4 (*DLL4*; Hs00184092_m1); and jagged canonical Notch ligand 1 (*JAG1*; Hs01070032_m1) (Applied Biosystems), according to manufacturer’s instructions. PCR was run with an Applied Biosystems 7500 fast real-time PCR system. Gene expression was normalized to the expression of *ACTB* (actin β), and relative gene expression (fold change) was calculated using the 2^−ΔΔCt^ method. Stable expression of *ACTB* as reference gene is presented in the Supplementary Information (supplementary Fig. S2).

### Quantification of specific ALP activity

ALP activity was measured and normalized to the DNA content of the respective samples. Samples were thawed and treated with 1% Triton X-100 in PBS for 50 min with an ultrasonication step for 10 min in between. ALP activity was determined colorimetrically by the cleavage of colorless *p*-nitrophenyl phosphate to yellowish *p*-nitrophenol and quantified by absorbance measurements at 405 nm using a spectrofluorometer infinite M200pro (Tecan Trading AG, Switzerland) [15]. DNA concentration of cell lysates was quantified using Quantifluor One dsDNA kit (Promega) according to manufacturer’s instructions.

### RANKL, OPG, and VEGF ELISA

Cell culture supernatants were analyzed with the following ELISA kits: Human TRANCE/RANKL/TNFS11 DuoSet ELISA no. DY626; Human Osteoprotegerin/TNFRSF11B DuoSet ELISA no. DY805 (both R&D Systems), and Human VEGF Standard TMB ELISA Development Kit no. 900-T10 (PeproTech) using recombinant human RANKL (78.1–5000 pg/mL), OPG (62.5–4000 pg/mL), or VEGF (125–2000 pg/mL) calibration curves.

### Statistics

Direct OB-HUVEC co-cultures and hOB cultures were performed in six independent experiments with four different donor combinations (see Materials and methods section *Cell culture*). For each medium condition, all samples of the individual experiments were seeded in triplicates for the measurement of ALP activity and ELISA-based quantification of protein secretion in cell culture supernatant, while gene expression was analyzed in two RNA samples generated from six replicates for each experimental group. Statistical differences in gene expression were calculated at the level of ΔCt values. Two-way analysis of variance (ANOVA), followed by Tukey’s multiple comparisons test was used to compare the experimental groups with different media compositions, with respect to the six individual experiments with different donor combination. Asterisks indicate significant differences between Ctrl, Dex, Dex VEGF10, Dex VEGF20, and VEGF20. Hashtags indicate a significant difference between VEGF20 and VEGF20 DAPT. All statistical analyses were performed with GraphPad Prism 10.0.

## Results

### Dex-induced impaired tube formation of HUVEC can be ameliorated by VEGF

**Fig. 2:**
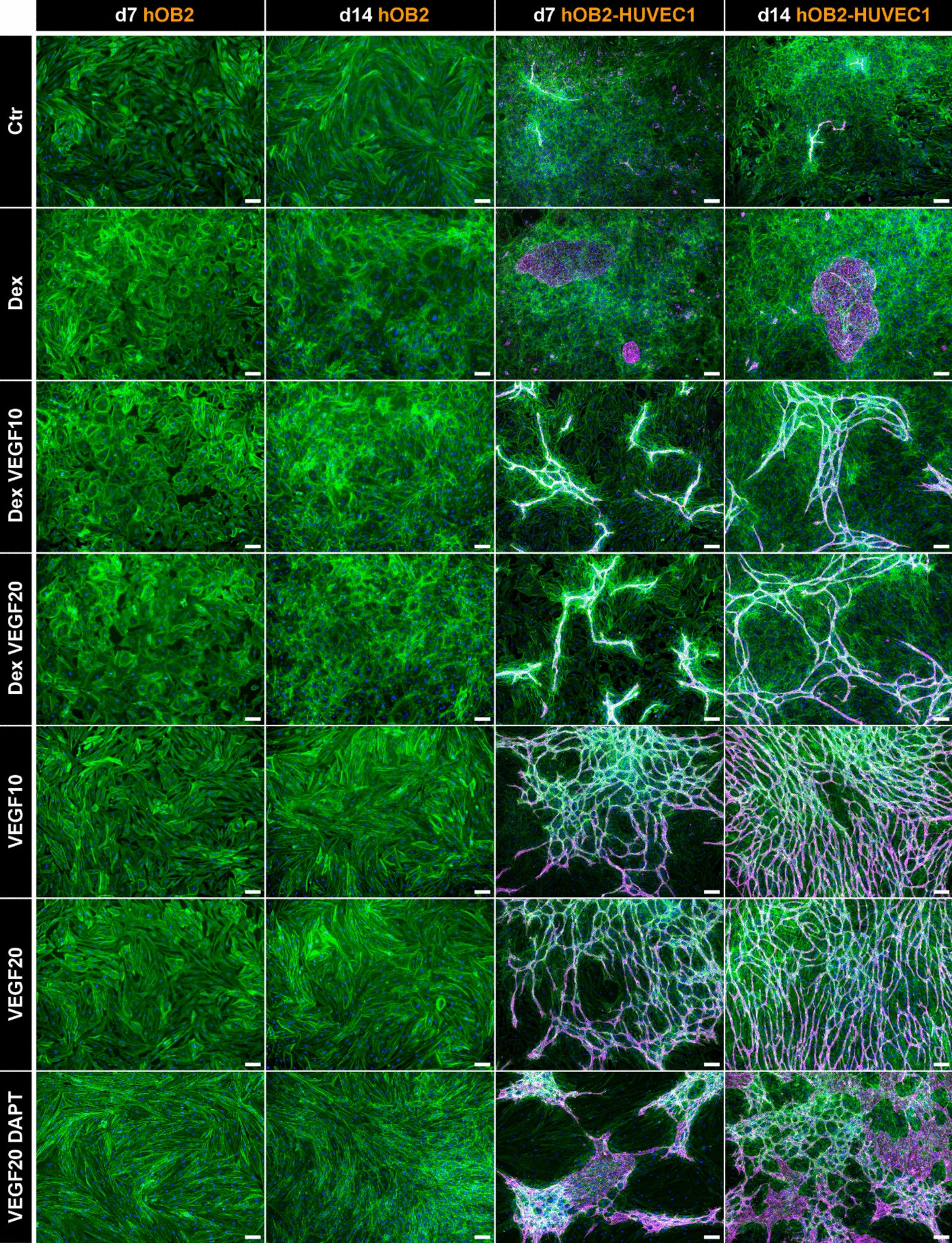
Fluorescence microscopic images of hOBs in monoculture and co-culture with HUVECs. Cells were imaged after 7/14 days of cultivation in different media. Cytoskeleton appears green (iFluor488 phalloidin), nuclei appear blue (DAPI), and CD31 appears magenta (Alexa Fluor 546). Scale bars represent 100 µm. Images of hOB2 and HUVEC1 are shown as examples because there were no differences in the cell morphology between experiments with different donor combinations

In control samples, without supplementation of Dex and VEGF, only few CD31-positive HUVECs, stained in magenta, were able to form tube-like structures on a dense hOB layer in direct co-cultures of hOBs and HUVECs (Fig. 2). Dex supplementation led to the formation of small HUVEC clusters. Only in media supplemented with VEGF, a dense tube network of HUVECs on top of hOBs was formed, already after 7 days of cultivation. HUVEC tube networks became denser with increasing VEGF concentration and over time. The combination of Dex and VEGF reduced the density of HUVEC tube networks. DAPT treatment impaired VEGF-induced tube formation of HUVECs and resulted in more cluster-like morphology of HUVECs than tube-formation. hOBs exhibited a cobblestone-like morphology in all media conditions, with a notable more rounded shape of hOBs in Dex-containing media.

Alizarin red staining indicated beginning mineralization in hOB cultures and hOB–HUVEC co-cultures (Supplementary Information: Supplementary Fig. S3). However, stronger alizarin red staining was observed in co-cultures, particularly in those treated with VEGF. The addition of Notch inhibitor DAPT decreased intensity of alizarin red staining compared with VEGF-containing groups.

### Dex and VEGF additively increase ALP in hOB–HUVEC co-culture but not in hOB monoculture, while DAPT inhibits ALP

**Fig. 3:**
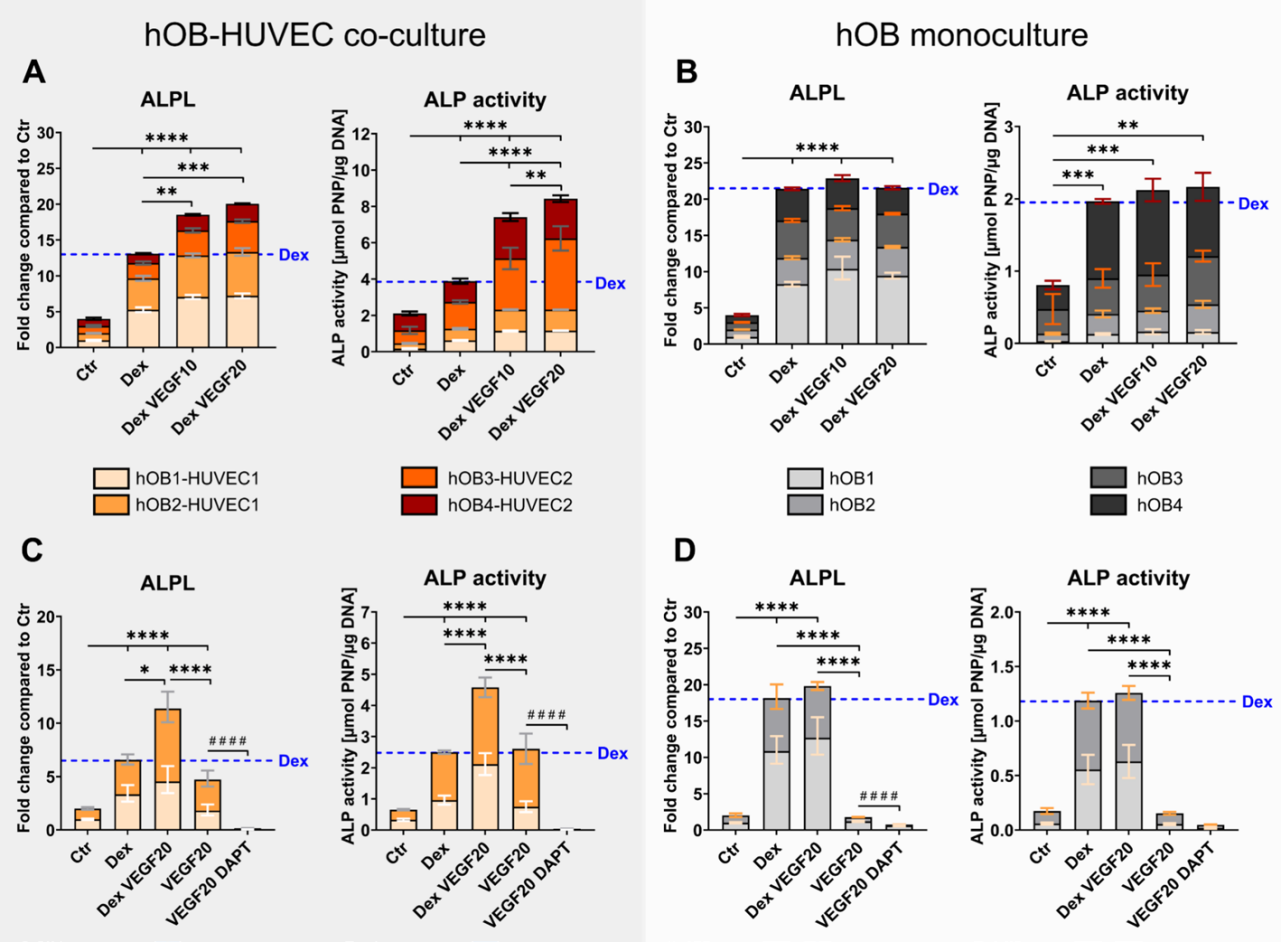
*ALPL* gene expression and ALP activity of hOB–HUVEC co-cultures (**A**, **C**) and hOB monocultures (**B**, **D**). Six independent experiments with four different donor combinations were conducted for 14 days in different media. Four individual experiments compare Ctr, Dex, Dex VEGF10, and VEGF20 treatment (**A**, **B**) and two additional experiments compare Ctr, Dex, DexVEGF20, VEGF20, and VEGF20 DAPT treatment (**C**, **D**). Gene expression is presented as fold change normalized to Ctr ± upper and lower limit (**A**, **B**: each experiment *n* = 6 per condition, in total *n* = 24 per condition; **C**, **D**: in total, *n* = 12 per condition). ALP activity is presented as mean value with standard deviation (**A**, **B**: each experiment *n* = 3 per condition, in total *n* = 12 per condition; **C**, **D**: in total *n* = 6 per condition). ^*^ *p* < 0.05; ^**^p < 0.01; ^***^p < 0.001; ^****^/^####^*p* < 0.0001. Asterisks indicate significant differences between Ctr, Dex, Dex VEGF10, Dex VEGF20, and VEGF20. Hashtags indicate significant difference between VEGF20 and VEGF20 DAPT

*ALPL* gene expression and ALP activity (*p* < 0.0001) increased in the presence of Dex in both hOB monoculture and hOB–HUVEC co-culture in all four independent experiments (Fig. 3A, B). When VEGF was supplemented additionally to Dex in co-culture, *ALPL* and ALP were significantly increased compared with only Dex-containing media. This effect was solely observed in hOB–HUVEC co-culture, not in hOB monoculture. A total of 10 ng/mL VEGF was sufficient to see this effect. In two additional experiments, VEGF was supplied without Dex and was shown to increase *ALPL* and ALP compared with the control in co-cultures, but not in hOB monocultures (Fig. 3C, D). Furthermore, both *ALPL* and ALP were significantly lower in VEGF medium compared with VEGF/Dex medium, suggesting an additive effect of Dex and VEGF in hOB–HUVEC co-cultures. Interestingly, the presence of the Notch inhibitor DAPT suppressed VEGF-induced *ALPL* expression and activity (Fig. 3C, D).

### VEGF increases RANKL/OPG ratio and ameliorates Dex-induced RANKL reduction in hOB–HUVEC co-cultures but not in hOB monocultures independent of Notch inhibitor DAPT

**Fig. 4:**
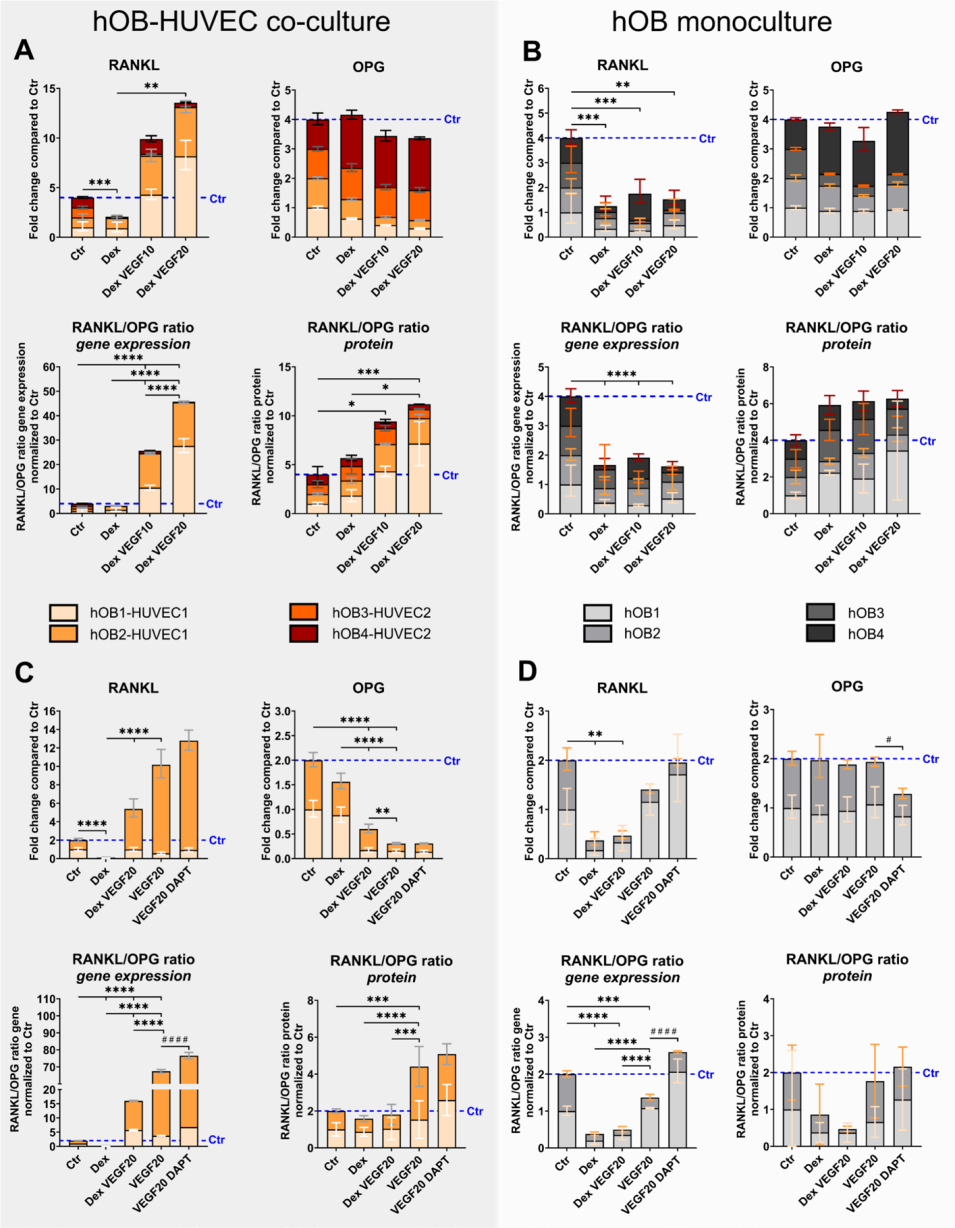
*RANKL*, *OPG* gene expression, and RANKL/OPG ratio of hOB–HUVEC co-cultures (**A**, **C**) and hOB monocultures (**B**, **D**). Six independent experiments with four different donor combinations were conducted for 14 days in different media. Four individual experiments compare Ctr, Dex, Dex VEGF10, and VEGF20 treatment (**A**, **B**) and two additional experiments compare Ctr, Dex, DexVEGF20, VEGF20, and VEGF20 DAPT treatment (**C**, **D**). Gene expression is presented as fold change normalized to control ± upper and lower limit (**A**, **B**: each experiment *n* = 6 per condition, in total *n* = 24 per condition; **C**, **D**: in total *n* = 12 per condition). RANKL/OPG protein ratio is presented as mean value with standard deviation (**A**, **B**: each experiment *n* = 3 per condition, in total *n* = 12 per condition; **C**, **D**: in total *n* = 6 per condition. ^*^/^#^*p* < 0.05; ^**^p < 0.01; ^***^p < 0.001; ^****^/^####^ *p* < 0.0001. Asterisks indicate significant differences between Ctr, Dex, Dex VEGF10, Dex VEGF20, and VEGF20. Hashtags indicate significant difference between VEGF20 and VEGF20 DAPT

The addition of Dex significantly reduced *RANKL* expression both in hOB monoculture and hOB–HUVEC co-culture (Fig. 4). However, the combination of Dex and 20 ng/mL VEGF significantly stimulated expression in hOB–HUVEC co-culture (Fig. 4A *p* < 0.01). In contrast,* RANKL* expression was downregulated in hOB monoculture in all Dex-containing media regardless of VEGF supplementation (Fig. 4B, D). In two additional experiments, VEGF was supplied without Dex and was shown to significantly increase *RANKL* expression compared with the Dex treated groups in co-culture (Fig. 4C *p* < 0.0001). In hOB monocultures, VEGF supplementation did not significantly increase *RANKL* expression compared with the control or Dex group (Fig. 4D). In addition, *OPG* expression significantly decreased in the presence of VEGF, but solely in co-culture, not in hOB monoculture (Fig. 4C, D). RANKL/OPG ratio on gene expression level was reduced in all Dex-containing media regardless of VEGF supplementation in hOB monocultures (Fig. 4B, D), whereas in co-culture, RANKL/OPG ratio increased in the presence of VEGF compared with the control (*p* < 0.0001) and compared with Dex group (*p* < 0.0001) (Fig. 4A, C). At protein level, RANKL/OPG ratio showed no significant difference between media conditions in hOB monoculture, but in hOB–HUVEC co-culture, VEGF stimulated RANKL/OPG protein ratio compared with the control and compared with Dex (Fig. 4A, C). Single RANKL and OPG protein concentrations in cell culture supernatants are provided in the Supplementary Information (Supplementary Fig. S4). Only in co-cultures, RANKL concentration in the cell culture supernatant was significantly higher in VEGF-containing media compared with Dex-treated groups (Supplementary Fig. S4A, C). The VEGF-induced increase of RANKL expression in parallel with reduction of OPG expression in hOB–HUVEC co-cultures was not significantly affected by DAPT treatment (Fig. 4C).

### *VEGF* expression is downregulated by Dex and VEGF, and the effect VEGF can be ameliorated by Notch inhibitor DAPT in hOB–HUVEC co-culture

**Fig. 5:**
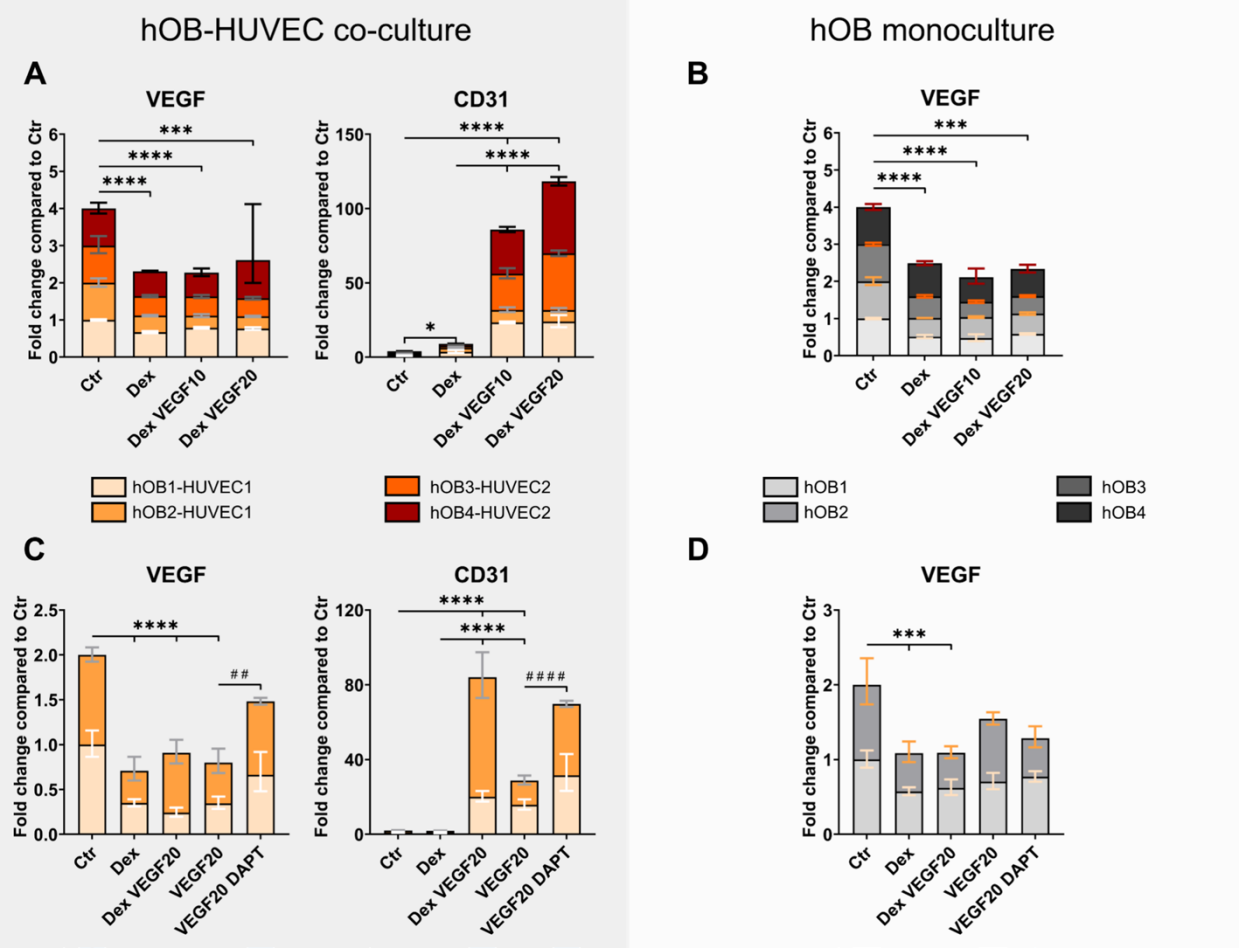
*VEGF* and *CD31* gene expression of hOB–HUVEC co-cultures (**A**, **C**) and hOB monocultures (**B**, **D**). Six independent experiments with four different donor combinations were conducted for 14 days in different media**.** Four individual experiments compare Ctr, Dex, Dex VEGF10, and VEGF20 treatment (**A**, **B**) and two additional experiments compare Ctr, Dex, DexVEGF20, VEGF20, and VEGF20 DAPT treatment (**C**, **D**). Gene expression is presented as fold change normalized to Ctr ± upper and lower limit (**A**, **B**: each experiment *n* = 6 per condition, in total *n* = 24 per condition; **C**, **D**: in total *n* = 12 per condition). ^*^p < 0.05; ^##^ *p* < 0.01; ^***^p < 0.001; ^****^/^####^*p* < 0.0001. Asterisks indicate significant differences between Ctr, Dex, Dex VEGF10, Dex VEGF20, and VEGF20. Hashtags indicate significant difference between VEGF20 and VEGF20 DAPT

In both hOB monoculture and hOB–HUVEC co-culture, *VEGF* gene expression was significantly downregulated in all Dex-containing media, with and without the addition of VEGF, (Fig. 5). In two additional experiments, VEGF was supplied without Dex, and *VEGF* expression was significantly reduced compared with the control group in co-culture (Fig. 5C), but not in hOB monoculture (Fig. 5D). In turn, the presence of DAPT increased *VEGF* expression compared with VEGF-containing media only in co-culture (Fig. 5C). Cell culture supernatants of hOB–HUVEC co-cultures contained a VEGF protein concentration of ~0.5 ng/mL per donor in control and Dex medium, and ~0.05 ng/mL in hOB monoculture. In VEGF-containing media, no further increase of the VEGF concentration that was initially added to the media was measurable (data not shown). *CD31* gene expression was strongly upregulated in the presence of VEGF in hOB–HUVEC co-culture (*p* < 0.0001) (Fig. 5A, C).

### VEGF induces upregulation of genes involved in NOTCH signaling (*NOTCH1*, *DLL4*, *JAG1*) in hOB–HUVEC co-cultures but not in hOB monocultures

**Fig. 6:**
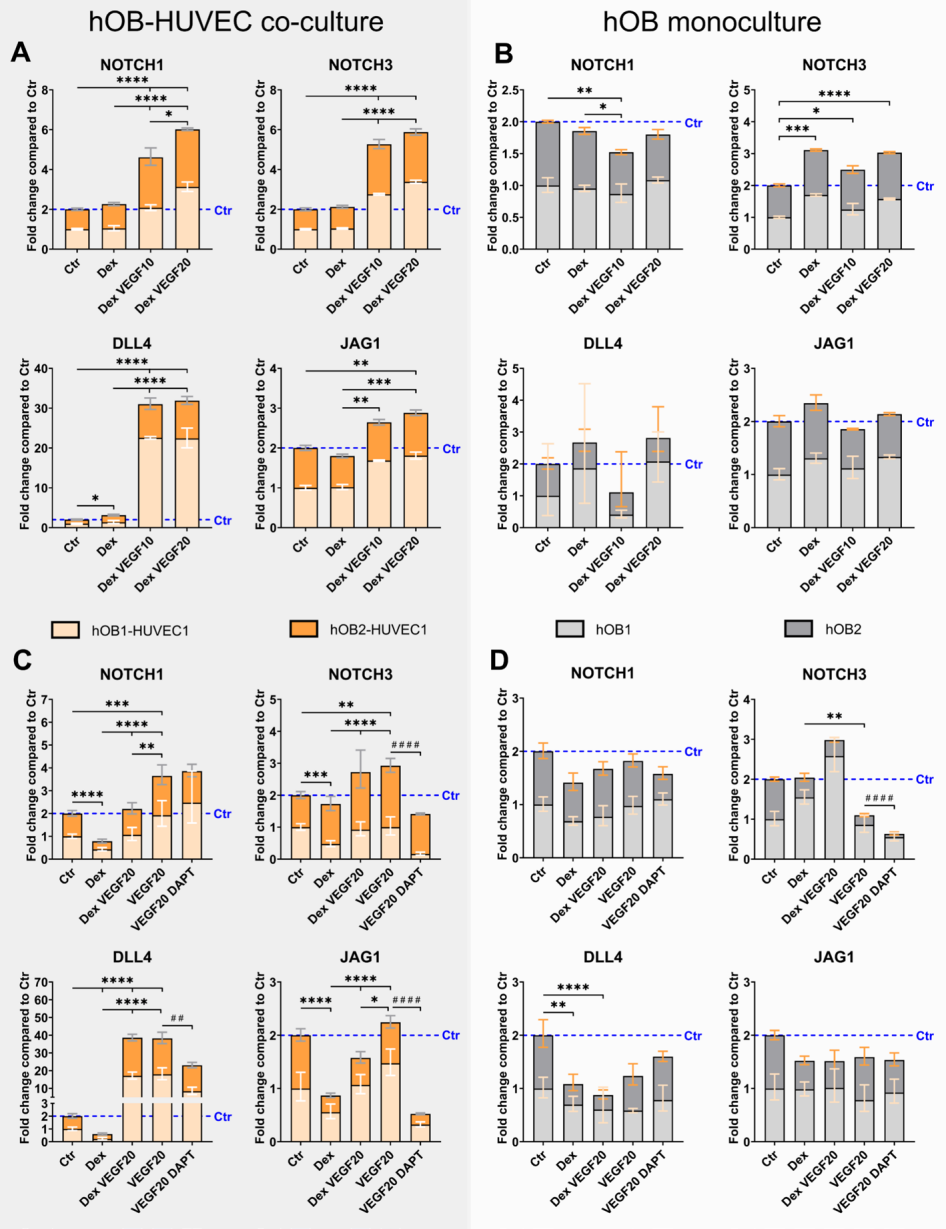
NOTCH gene expression of hOB–HUVEC co-cultures (**A**, **C**) and hOB monocultures (**B**, **D**). Four independent experiments with two different donor combinations were conducted for 14 days in different media comparing Ctr, Dex, DexVEGF20, VEGF20, and VEGF20 DAPT treatment. Gene expression is presented as fold change normalized to Ctr ± upper and lower limit (each experiment *n* = 6 per condition, in total *n* = 12). ^*^p < 0.05; ^**^p < 0.01; ^***^/^###^ *p* < 0.001; ^****^/^####^ *p* < 0.0001. Asterisks indicate significant differences between Ctr, Dex, Dex VEGF10, Dex VEGF20, and VEGF20. Hashtags indicate significant difference between VEGF20 and VEGF20 DAPT

Gene expression of *NOTCH1*, *NOTCH3*, *JAG1*, and *DLL4* was significantly upregulated in hOB–HUVEC co-cultures in VEGF-containing media compared with control and Dex-containing media in two independent experiments (Fig. 6A). Strongest gene expression in the presence of VEGF in co-culture was observed for *NOTCH1* and *DLL4*. In contrast, *NOTCH1*, *JAG1*, and *DLL4* were not upregulated in hOB cultures after VEGF treatment (Fig. 6B). *NOTCH2* gene expression was not induced by VEGF in either hOB–HUVEC co-culture or hOB culture (Supplementary Information: Supplementary Fig. S5). Only *NOTCH3* gene expression showed a significant increase in response to VEGF in hOB monocultures of two experiments (Fig. 6B). In two additional experiments with only VEGF-containing media (Fig. 6C, D), significant stimulation of *NOTCH1*, *NOTCH3*, and *DLL4* by VEGF compared with VEGF-free control and Dex treatment was detected again only in hOB–HUVEC co-cultures (Fig. 6C, D). The presence of NOTCH inhibitor DAPT reduced gene expression of *NOTCH3*, *DLL4*, and *JAG1* in VEGF-treated co-culture compared with VEGF treatment alone (Fig. 6C). In hOB monoculture, expression of NOTCH-related genes was not significantly induced under any condition compared with the control (Fig. 6D).

## Discussion

The interaction between endothelial cells and osteoblasts plays a key role in bone regeneration. The present study focused on the crosstalk between these cell types under the influence of the glucocorticoid dexamethasone, VEGF, and their combination. Major differences between hOB cultures and hOB–HUVEC direct co-cultures indicated a significant influence of endothelial cells on ALP and RANKL/OPG ratio of OBs, with strong involvement of the Notch signaling pathway in the regulation of ALP.

**Fig. 7:**
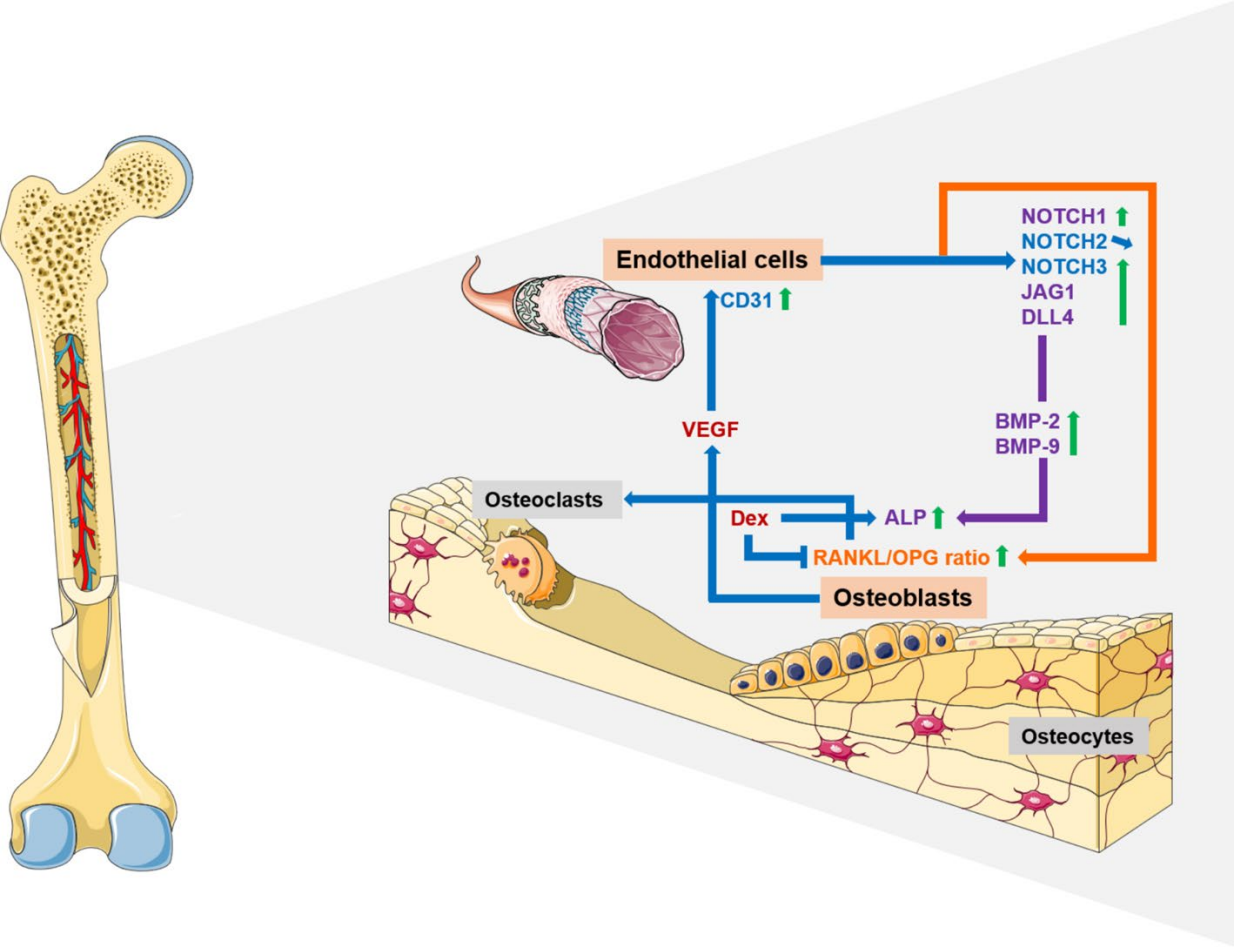
Signaling pathway visualizing the crosstalk between bone cells and endothelial cells via Notch and BMP signaling. The effect of VEGF and Dex (colored in red) was analyzed in hOB compared with hOB–HUVEC co-cultures. VEGF promoted endothelial cell survival and functionality, resulting in upregulation of Notch signaling in hOB–HUVEC co-culture. Following downstream, ALP of hOBs was upregulated, possibly due to induction of BMP-2 and BMP-9 via NOTCH1-JAG1/DLL4 axis (colored in violet). RANKL/OPG ratio increased in the presence of VEGF in co-culture, independent from Notch signaling (colored in orange). Dex treatment stimulated ALP of hOBs and decreased RANKL/OPG ratio. This figure was created with modified illustrations from Servier Medical Art

ALP is a well-established early osteogenic marker. Increased ALPL gene expression and corresponding ALP activity in response to Dex treatment in hOB monocultures and hOB–HUVEC co-cultures (Fig. 3) demonstrated the pro-osteogenic effect of Dex on *ALPL* gene expression [16, 17] and ALP activity [18] of OBs. VEGF had no effect on *ALPL* gene expression and ALP activity in hOB monocultures. In contrast, ALP in hOB–HUVEC co-culture was similarly stimulated in VEGF-containing media compared with Dex-containing media, and significantly upregulated in media containing both Dex and VEGF. These results suggest a direct stimulatory impact of HUVEC, supported by VEGF treatment, on *ALPL* gene expression and ALP activity of OBs. It has been postulated before, that endothelial cells are able to stimulate osteogenic differentiation. Direct cell–cell contact between endothelial and osteoprogenitor cells was shown to be necessary for this stimulation [19]. Strongly increased ALP activity in co-cultures of osteoblast-like MG-63 human osteosarcoma cell line with HUVEC compared with MG-63 monoculture was also reported previously [5, 20] and confirmed the results in the present co-culture experiments with primary human cells. In addition, paracrine signaling between HUVEC and OB in a non-contact co-culture also increased ALP activity of OBs compared with OB monocultures [21]. In conclusion, externally added VEGF indirectly influences OB differentiation through actions on HUVEC via endothelial cell-derived osteogenic factor(s), which are yet still undefined. However, our study suggests a role of Notch signaling in endothelial cells that induced osteogenic differentiation of OBs (Fig. 6). The Notch signaling pathway regulates cell-to-cell signal transduction by coordinating cell proliferation, differentiation, and apoptosis. Canonical Notch signaling pathway is mainly composed of four Notch receptors (Notch1–4) and five Notch ligands (Jagged1, 2 and Delta-like 1, 3, and 4) in mammals [22, 23]. Although Notch receptors have a very similar structure, they are not redundant, and various receptor-ligand combinations specifically transmit signals [24]. VEGF induces NOTCH1 and its ligand DLL4 in endothelial cells [25, 26], which was confirmed in our hOB–HUVEC co-culture study. Following downstream, NOTCH1 is able to initiate bone morphogenetic protein (BMP)-9 induced stimulation of ALP in mesenchymal stem cells (mouse embryo fibroblasts) [27]. Furthermore, Notch signaling in OBs, involving NOTCH1 and its ligands DLL1 and JAG1 enhances BMP-2-induced osteogenic differentiation of OBs, including ALP [28–30]. Since Notch gene expression (*NOTCH1*, *DLL4*, *JAG1*) was not upregulated in hOB monoculture but in hOB–HUVEC co-culture, we hypothesize that the increased *ALPL* gene expression and ALP activity of hOBs in co-culture with HUVEC in the presence of VEGF is regulated by Notch signaling in endothelial cells, specifically by the interplay of NOTCH1 and DLL4/JAG1, and BMP signaling, thereby triggering osteogenic differentiation of hOBs (Fig. 7). This hypothesis is supported by the inhibition of VEGF-induced *ALPL* expression and activity in the presence of the Notch inhibitor DAPT (Fig. 3). Notch proteins are substrates for γ-secretase, and DAPT is a γ-secretase inhibitor that indirectly suppresses Notch signaling by preventing the release and transcriptional activity of the Notch intracellular domain (NICD) [31]. Therefore, DAPT inhibits not necessarily the expression of all Notch receptors and ligands, but rather inhibits the transcription of target genes downstream of NICD activity, explaining why gene expression of Notch receptors and ligands itself were not inhibited by DAPT treatment in this study. The presented results are consistent with recent findings demonstrating induction of osteogenic differentiation by Notch signaling in endothelial cells, depending on direct contact between osteoblasts and HUVEC [32]. Since VEGF, rather than Dex treatment, stimulated Notch signaling (Fig. 6), we only added the Notch inhibitor DAPT to VEGF-supplemented media to analyze the role of the Notch signaling pathway in our study.

The RANK–RANKL–OPG system is a complex signaling pathway playing an important role in bone metabolism. Bone-resorbing osteoclasts derive from progenitor cells of the monocyte/macrophage family in the presence of two requisite cytokines: macrophage colony stimulating factor (MCSF) and RANKL [33]. A known side effect of long-term Dex administration as anti-inflammatory drug is osteoporosis and therefore the loss of bone density due to increased bone resorption and insufficient bone formation. However, the exact mechanism behind Dex-induced osteoporosis is not comprehensively known. Many studies address the effect of glucocorticoids such as Dex on RANKL-induced osteoclastogenesis with partially contradictory results that point to the complex mechanism of action of Dex on the RANK–RANKL–OPG system: A number of publications demonstrate upregulation of *MCSF *and *RANKL* gene expression as well as downregulation of *OPG* gene expression in response to glucocorticoid treatment of stromal and osteoblastic cells [34, 35]. In addition, Dex increased the RANKL/OPG ratio of SaOS-2 cells [36]. In vivo studies with Dex-treated rats confirmed the abovementioned in vitro studies by increased *RANKL *and decreased *OPG* gene expression in the rat femur tissue [37]. In contrast, Kim et al. did not observe significant differences of *RANKL* and *OPG* gene expression between Dex-treated co-cultures of mouse calvarial osteoblastic cells and bone marrow cells and a Dex-free control [38]. RANKL/OPG ratio of MC3T3-E1 cells and murine osteocyte-like MLO-Y4 cells was also not significantly changed when cells were cultivated in Dex-containing media [6]. Moreover, Dex treatment of mesenchymal stem cells isolated from the jaw periosteum lead to the tendency of reduced *RANKL* expression [39]. In the present study with primary human OBs and HUVEC, we showed for the first time a significant downregulation of *RANKL* gene expression in response to Dex treatment in hOB monoculture and hOB–HUVEC co-culture with six individual experiments with four different donor combinations (Fig. 4). Upregulation of *RANKL* gene expression as well as RANKL/OPG ratio in the presence of Dex was only observed in hOB–HUVEC co-cultures when VEGF was additionally added to the medium (Fig. 4A, C). Our results suggest a critical role of endothelial cells, supported by VEGF, on the RANK–RANKL–OPG system in bone tissue. Therefore, the interplay between bone cells and endothelial cells, present in close proximity in bone tissue, appears to be also important for the regulation of osteoclastogenesis and thus for the mechanism of Dex-induced osteoporosis. This hypothesis is supported by in vivo studies showing increased *RANKL* gene expression [37, 40] and suppressed OPG production in response to glucocorticoids in rat [37] or mice femur tissue [41] as well as zebrafish larvae [40]. The abovementioned inconsistent in vitro findings in studies regarding the effect of Dex treatment on the RANKL/OPG ratio may be explained by the use of different cell types and cell lines as well as variances in the concentration and duration of Dex treatment. However, the use of primary human osteoblasts and endothelial cells in our co-culture study, in comparison with primary cells or even cell lines of animals, most closely resembles the human in vivo situation. However, quantified RANKL protein concentrations were generally low, ranging from 20 to 150 pg/mL, depending on the individual experiment. This could be explained by the fact that only secreted, soluble RANKL was quantified in the cell culture supernatants. It has been previously reported that active RANKL is typically membrane-bound [42, 43] and therefore cannot be detected by ELISA measurements of supernatants. In contrast, gene expression data rely on RNA extracted from cell lysates, including cells in the presence of the membrane-bound RANKL fraction.

The stimulation of Notch signaling in response to VEGF was discussed above in the context of VEGF-induced *ALPL* expression in hOB–HUVEC co-culture. Our results suggest a regulatory role of endothelial cells not only on *ALP*L expression of hOBs but also on their RANKL/OPG ratio. The participation of the Notch pathway in osteoclastogenesis is still controversial: Canalis et al. described the induction of RANKL levels in response to NOTCH3 activation in OBs and osteocytes [44]. In addition, DLL4 significantly increased NOTCH3 signaling [45], which is consistent with parallel upregulation of *DLL4* and *NOTCH3* gene expression in hOB–HUVEC co-cultures (Fig. 6). NOTCH1 activation also directly modulates RANKL-induced osteoclastogenesis. However, constitutive activation of NOTCH1 signaling induced a block in osteoclast differentiation [46]. This regulation is proposed to be directly dependent on the cell stage of osteoclast progenitors and is mainly involved in the early phases of human osteogenesis. NOTCH2 is thought to stimulate osteoclastogenesis by inducing RANKL in OBs [47], but, since *NOTCH2* was not induced in our study (Supplementary Fig. S1), the observed stimulation of the RANKL/OPG ratio in hOB–HUVEC co-culture must be caused by other reasons. The presence of Notch inhibitor DAPT did not reduce the increased RANKL/OPG ratio in co-cultures treated with VEGF, allowing the conclusion that upregulation of RANKL as well as downregulation of OPG in hOB–HUVEC co-culture is independent from Notch signaling in endothelial cells (Fig. 4).

Only in VEGF-containing media HUVECs were able to form tube-like structures (Fig. 2). This essential role of VEGF in endothelial cell survival and functionality, facilitated by binding to its receptor VEGF-R on the surface of endothelial cells, has been well studied [3, 7]. The amount of VEGF, which was produced by hOBs, was not sufficient to induce tube formation in hOB–HUVEC co-cultures. Dex treatment impaired tube formation in HUVECs (Fig. 2) and decreased *VEGF* expression in hOBs (Fig. 5). It has been shown before that Dex inhibits tube formation of endothelial cells [48, 49]. Langendorf et al. furthermore demonstrated a decrease in *CD31* expression and a reduced *VEGF* expression in co-cultures of HUVECs with myoblasts in response to Dex [50]. Glucocorticoid-induced decrease in *VEGF* expression has also been demonstrated in a minipig study, revealing significantly lower VEGF levels in the bones of glucocorticoid-treated animals [51]. In addition, glucocorticoid administration reduced local vascularization by decreased hypoxia-inducible factor 1-α (*HIF-1α*) and *VEGF* expression in the femoral head of mice [52]. A notable transformation of hOB morphology in the presence of Dex (Fig. 2) to a more cuboidal cobblestone-like shape compared with Dex-free media indicated the well-known osteogenic effect of Dex on OB differentiation [53]. These findings confirm both the inhibitory effect of Dex on angiogenesis as well as its stimulatory effect on osteogenesis.

## Conclusions

The interaction between bone and endothelial cells is significant for bone remodeling. The present study provides new important implications for understanding the mechanisms that facilitate and control the communication between these cell types. First, the presence of endothelial cells supported by VEGF-induced *ALP*L gene expression and activity of bone-forming OBs as well as their RANKL/OPG ratio (Fig. 7). Second, Notch signaling is involved in the regulation of ALP through the NOTCH1-DLL4/JAG1 axis. Third, RANKL/OPG ratio is reduced after Dex treatment, but stimulated in the presence of VEGF, only in hOB–HUVEC co-cultures and independently of Notch signaling. Therefore, we suggest that endothelial cells may play an important role in the mechanism of Dex-induced osteoporosis via yet unknown endothelial cell-derived factor(s). We demonstrated the importance of endothelial cells in bone remodeling and identified the Notch signaling pathway as one of the key regulators. The present study highlights the applicability of more complex, multicellular in vitro models using primary human cells to study the processes behind bone metabolism.

## Supplementary Information

Supplementary file 1.

## Data Availability

All data reported within this article are available in supplementary files or will be made available upon request.

## Abbreviations

AAP: Ascorbic acid-2-phosphate
ALP: Alkaline phosphatase
BMP: Bone morphogenetic protein
CD31/PECAM1: Platelet endothelial cell adhesion molecule
DAPT: N-[N-(3,5-difluorophenacetyl)-l-alanyl]-S-phenylglycine t-butyl ester
Dex: Dexamethasone
DLL: Delta-like canonical Notch ligand
EBM: Endothelial cell basal medium
EGM: Endothelial cell growth medium
HIF: Hypoxia inducible factor
HUVEC: Human umbilical vein endothelial cells
JAG: Jagged canonical Notch ligand
NOTCH: Neurogenic locus Notch homolog protein
OB: Osteoblast
OPG: Osteoprotegerin
PS: Penicillin–streptomycin
RANKL: Receptor activator of nuclear factor κB ligand
VEGF: Vascular endothelial growth factor
β-GP: β-glycerophosphate
