# Supplementary material for: Crosstalk between endothelial cells and osteoblasts stimulates ALP via Notch signaling and RANKL/OPG ratio independently of Notch signaling in vitro
Source: Cell Mol Biol Lett. 2025 Oct 2;30:108. doi: 10.1186/s11658-025-00793-9 (PMC12490079; doi:10.1186/s11658-025-00793-9)
Supplement: Supplementary file 1 [file 11658_2025_793_MOESM1_ESM.docx]

**Supplementary Information**

**Crosstalk between endothelial cells and osteoblasts stimulates ALP via Notch signaling and RANKL/OPG ratio independently of Notch signaling in vitro**

Katharina Wirsig^1^, Nina Bürger^1^, Anne Bernhardt^1^*

^1^Centre for Translational Bone, Joint- and Soft Tissue Research, Faculty of Medicine and University Hospital, TUD University of Technology, Fetscherstraße 74, 01307 Dresden, Germany

*Corresponding author: Dr. Anne Bernhardt

[Anne.bernhardt@tu-dresden.de](mailto:Anne.bernhardt@tu-dresden.de), Tel.: +49 351 458-6692, Fax: +49 351 458-7210


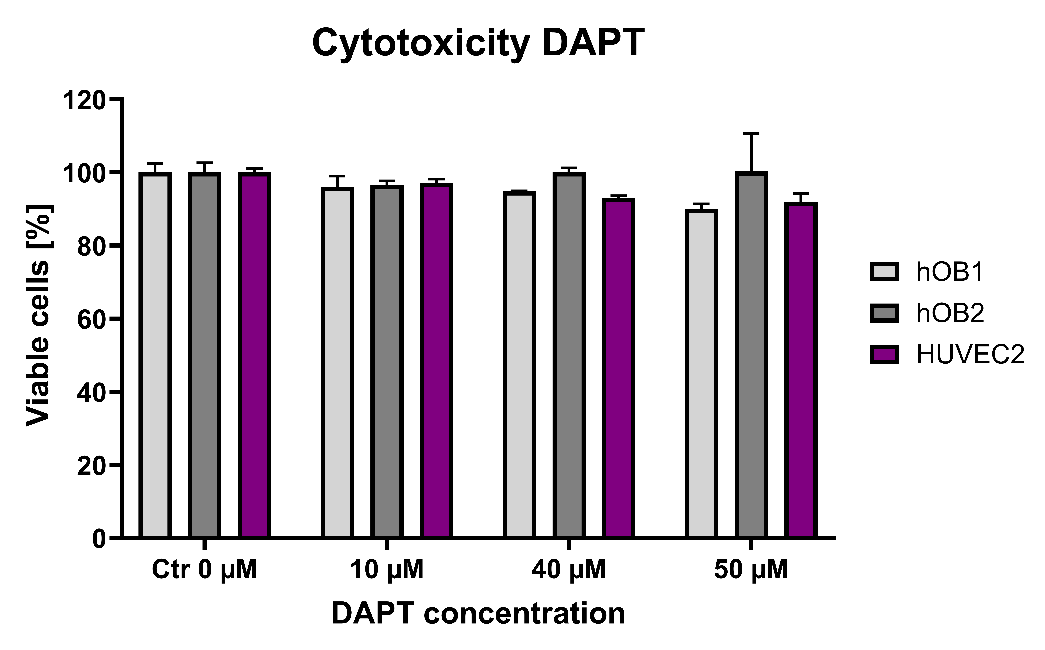


Fig. S1 Viability of hOB and HUVEC in response to different DAPT concentrations (10 µM, 40 µM, 50 µM; each donor and concentration n=3). Bars present average percentage of viable cells with standard deviation.


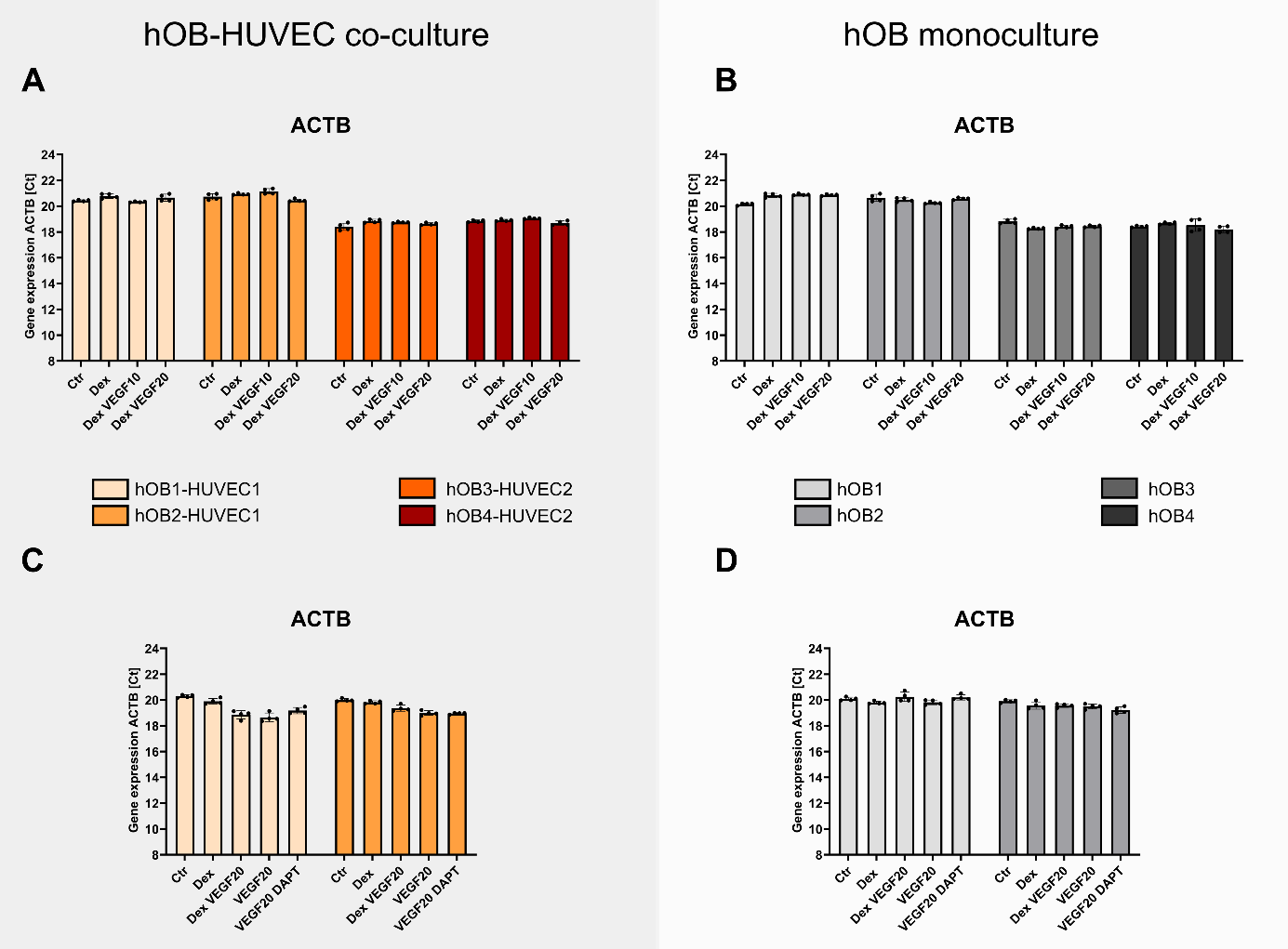


Fig. S2 Gene expression of ACTB in hOB-HUVEC co-cultures (A, C) and hOB monocultures (B,D). Six independent experiments with four different donor combinations were conducted for 14 days in different media. Four individual experiments compare Ctr, Dex, Dex VEGF10, and VEGF20 treatment (A, B) and two additional experiments compare Ctr, Dex, DexVEGF20, VEGF20, and VEGF20 DAPT treatment (C, D). Gene expression is presented as ΔCt value. Each experiment n=6 per condition.


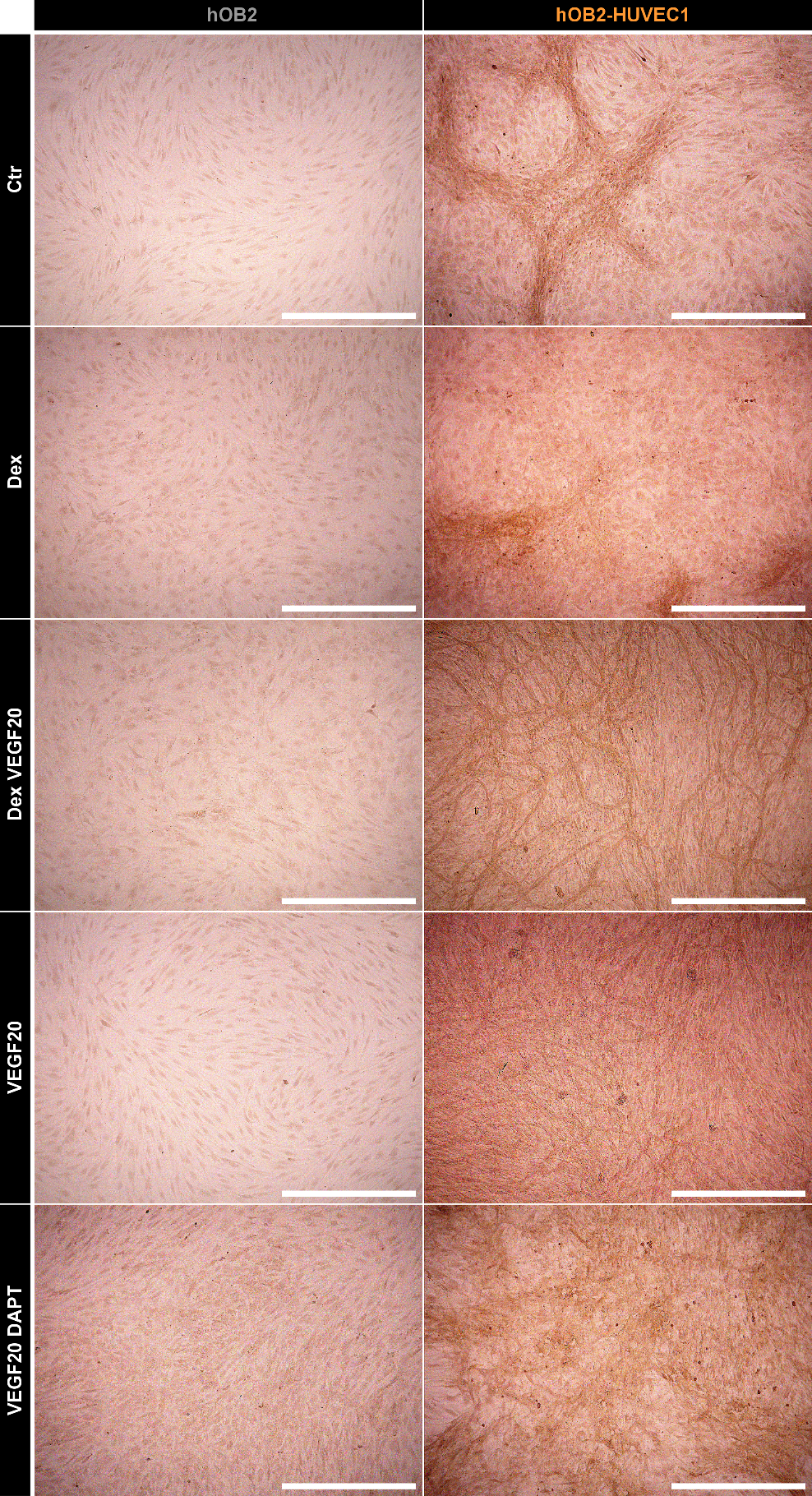


Fig. S3 Alizarin red staining of hOB-HUVEC co-cultures and hOB monocultures after 14 days cultivation in different media. The impact of Dex, VEGF, their combination or VEGF in the presence of Notch inhibitor DAPT on mineralization was tested. Scale bars represent 500 µM.


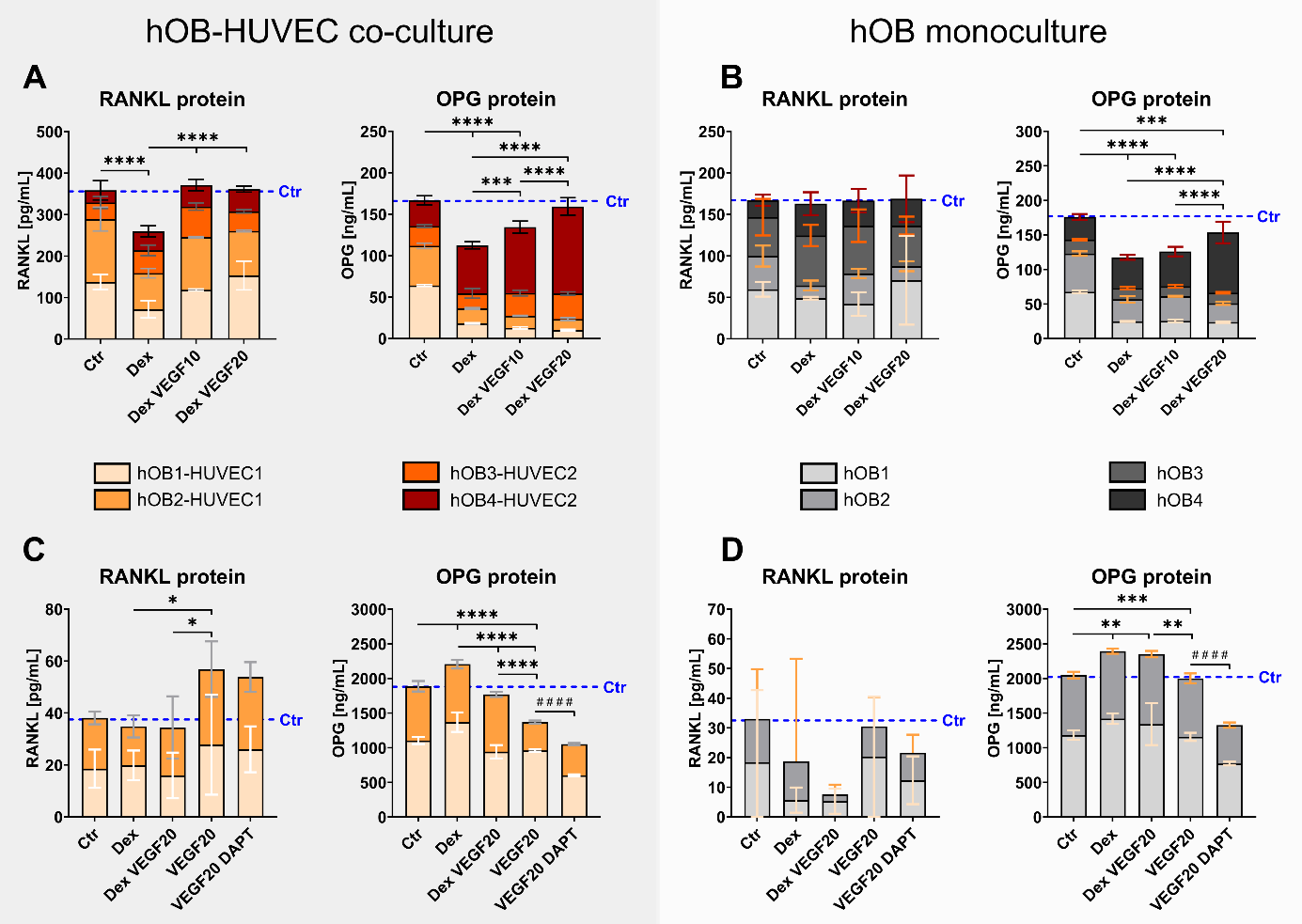


Fig. S4 RANKL and OPG protein concentration in cell culture supernatants of hOB-HUVEC co-cultures (A, C) and hOB monocultures (B, D). Six independent experiments with four different donor combinations were conducted for 14 days in different media. Four individual experiments compare Ctr, Dex, Dex VEGF10, and VEGF20 treatment (A, B) and two additional experiments compare Ctr, Dex, DexVEGF20, VEGF20, and VEGF20 DAPT treatment (C, D). Gene expression is presented as fold change normalized to Ctr ± upper and lower limit (A, B: each experiment n=3 per condition, in total n=12 per condition; C, D: in total n=6 per condition). *p < 0.05; **p < 0.01; ***p < 0.001; ****/^####^ p < 0.0001. Asterisks indicate significant differences between Ctr, Dex, Dex VEGF10, Dex VEGF20 and VEGF20. Hashtags indicate significant difference between VEGF20 and VEGF20 DAPT.


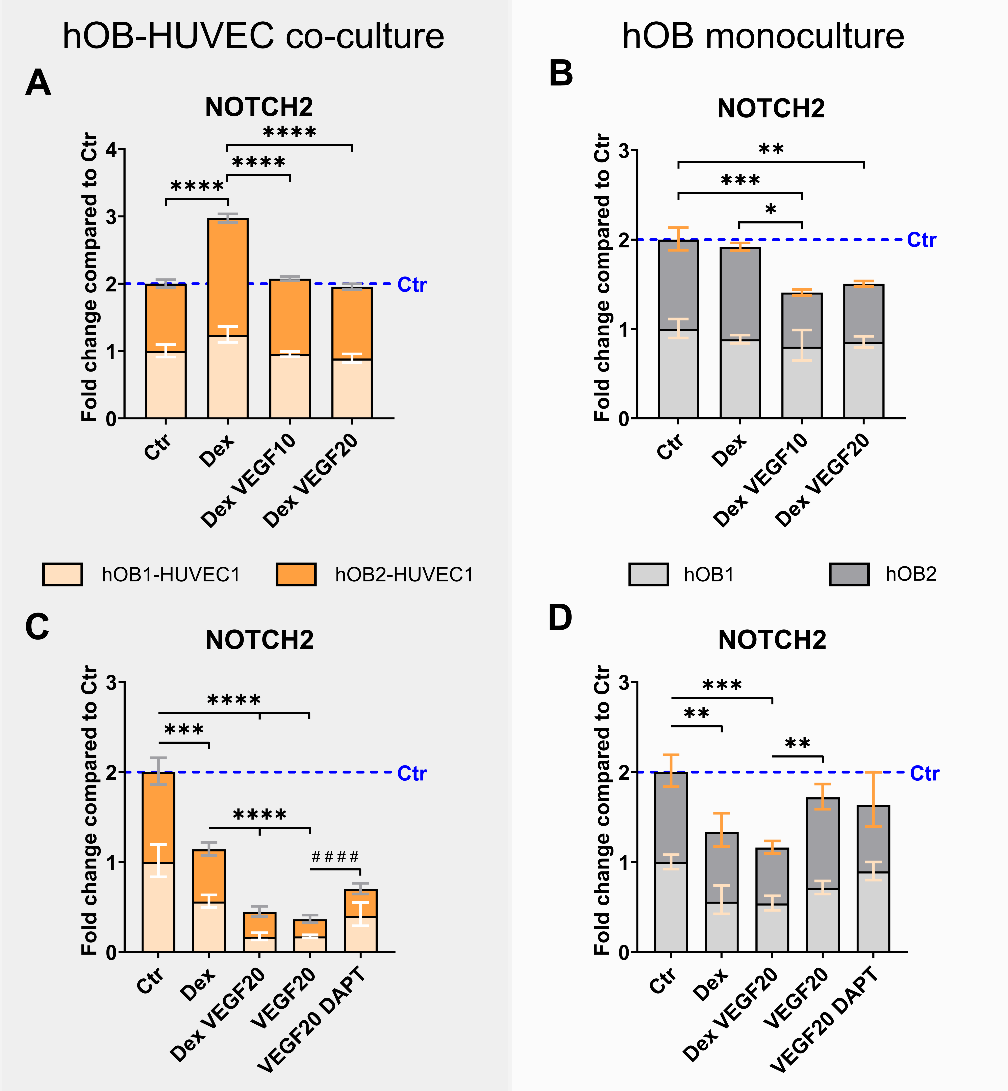


Fig. S5 NOTCH2 gene expression of hOB-HUVEC co-cultures (A, C) and hOB monocultures (B, D). Four independent experiments with two different donor combinations were conducted for 14 days in different media. Two individual experiments compare Ctr, Dex, Dex VEGF10, and VEGF20 treatment (A, B) and two additional experiments compare Ctr, Dex, DexVEGF20, VEGF20, and VEGF20 DAPT treatment (C, D). Gene expression is presented as fold change normalized to Ctr ± upper and lower limit (each experiment n=6 per condition, in total n=12 per conditionn). *p < 0.05; **p < 0.01; ***p < 0.001; ****/^####^ p < 0.0001. Asterisks indicate significant differences between Ctr, Dex, Dex VEGF10, Dex VEGF20 and VEGF20. Hashtags indicate significant difference between VEGF20 and VEGF20 DAPT.
